# An assessment of public experiences and expectations with physicians: a cross sectional study from Karachi, Pakistan

**DOI:** 10.1186/s12913-023-10519-2

**Published:** 2024-01-18

**Authors:** Hammad Atif Irshad, Muhammad Umar Mahar, Arshia Jahangir, Syed Roohan Aamir, Muhammad Abdullah Jamil, Khizer Ahsan, Maliha Taufiq, Abdullah Ahmed, Shilpa Golani, Sehba Amir, Wasila Gul Kakar, Abida Ali, Asaad Ahmed Nafees

**Affiliations:** 1 Medical College, Aga Khan University, Karachi, Pakistan; 2 Department of Community Health Sciences, Aga Khan University, Karachi, Pakistan

**Keywords:** Public health, Health services, Healthcare experiences, Patient-Centered care, Public expectations

## Abstract

**Background:**

Although physicians are highly regarded members of society, patients are not always satisfied with their care, suggesting a mismatch between the public’s expectations and reality. Thus, the aim of this study was to determine the public’s expectations regarding roles and responsibilities of a physician, to assess patient experiences, and to evaluate factors associated with the two outcomes.

**Methods:**

A cross-sectional study was conducted via face-to-face structured interviews from July 14th to August 2nd, 2023, in Karachi, Pakistan. The study sample comprised 424 consenting adults enrolled by visiting public spaces (malls, parks, hospitals, and residential areas). A modified version of ‘Exceptionally Good Doctor Likert scale’, and ‘Patient Picker-15’ (PPE-15) questionnaires was used. The Likert and PPE-15 sections were scored through pre-decided criteria for expectations and experience, respectively, and categorized using a median cut-off into high and low expectations and negative and positive experiences, respectively for simple and multivariable logistic regression.

**Results:**

A median score of 30.5/ 34 (IQR = 3.3) was found for expectations and 4/ 14 (IQR = 4) for experiences. Significant factors associated with expectations were older age groups (OR = 4.54 [1.18–17.50]) and higher monthly household incomes (0.40 [0.20–0.79]), while the odds of negative experiences were lower after visits to emergency departments (0.38 [0.18–0.84]) and private health care centers (0.31 [0.13–0.70]).

**Conclusion:**

These results suggest that the public has high expectations from physicians, however their experiences are not always positive. Initiatives to develop a patient-centric ethos are needed for which we outline recommendations to both the public and physicians, respectively.

**Supplementary Information:**

The online version contains supplementary material available at 10.1186/s12913-023-10519-2.

## Introduction

The World Health Organization (WHO) underscores patient-physician relationship as the most fundamental contributor of optimal clinical outcomes [1]. A comprehensive investigation into the characteristics of both doctors and patients within the context of primary healthcare in low- and middle-income countries (LMICs) pointed out that while clinical expertise and competence are perceived as fundamental physician traits, patients accorded greater value to verbal and non-verbal communication over clinical attributes during consultations [2]. Consequently, the alignment of health care practices in a way that patient’s experiences can meet their expectations has gained recognition as one of the key pillars of quality primary medical care [3].

One in six physicians in a study conducted in Karachi, Pakistan, reported experiencing physical violence in the year preceding the study [4]. Jamali et al. reported unreasonable and unmet expectations and ineffective communication between the physician and patient as the leading contributors of physician-targeted violence [5]. Furthermore, studies have highlighted inadequate infrastructure and considerable unmet need for physicians give rise to subpar quality of primary healthcare facilities resulting in negative experiences of individuals that further widen the gap between patient’s expectation and experience [6]. However, due to the scarcity of studies highlighting the determinants of public’s dissatisfaction with medical practitioners, there’s an urgent need to explore ineffective rapport building and inadequate healthcare practices with the need to improve doctor-patient dynamics [7].

Numerous studies emphasize the importance of understanding expectations from their healthcare providers and contrasting these with their real experience, allowing us to improve the areas prioritized by the patients [8, 9]. The existing body of research regarding public’s expectations predominantly originates from contexts outside of LMICs, particularly Pakistan, where the highlighted values might not accurately reflect those held within our own population. Moreover, there is an urgent need to address the growing violence, misconceptions, mistrust, and general lack of confidence in the public towards physicians [10]. Therefore, to have a better-quality evaluation of mismatch between patients’ expectations and experiences and to identify factors affecting the poor interpersonal doctor-patient relationship, we identified the need to conduct a baseline evaluation of overall public’s expectations about physician’s responsibilities and experience within the healthcare system. Thus, the aim of this study was to determine the expectations of the public regarding the role and responsibilities of a physician and to assess patient experiences, and to evaluate factors affecting the two.

## Materials & methods

### Study design and setting

This was a cross-sectional study conducted amongst those residing in Karachi, Pakistan. Individuals who were at least 18 years old and were proficient in Urdu or English were included in the study. Healthcare and allied health workers were excluded to ensure that expectations and experiences of the public outside of healthcare were gauged.

A total number of 424 participants were interviewed by trained interviewers. The sample size was determined by considering relevant previous studies from which negative experiences had a prevalence of 73% and 77% prevalence of high scores for exceptionally good doctors [11, 12]. Considering these findings, the sample size calculated using OpenEpi was 384 participants, which upon adjusting for 10% margin of inaccurate responses led to the expected recruitment of 424 participants [13].

### Study questionnaire

Due to the absence of a single questionnaire which assessed both experiences and expectation, the questionnaire utilized in this study was a composite of a previously utilized tools, the Patient Picker Experience-15 (PPE-15) [11], a validated tool which has been used in several countries to measure experiences and the Exceptionally Good Doctor Survey [12], a questionnaire that has been used in various population groups, which we modified to assess expectations.

The final questionnaire (Supplementary File 1) consisted of four components. The first section contained questions regarding sociodemographic characteristics of the participants such as participants’ age, sex, education level, monthly income, area of residence, employment status, occupation and known comorbid. The second section used a modified version of the Likert scale from the Exceptionally Good Doctor Survey [12] to evaluate the expectation of a participant for a given attribute of a doctor. This ranged from the presence of an attribute ‘all the time’ to ‘Never’. In this case, responses were scored on a scale, with 1 point awarded for every instance of “all the time” being selected, 0.75 for “most of the time”, 0.5 for “sometimes”, 0.25 for “rarely”, and 0 for “never”. The maximum possible score for expectations was 34 points.

The third section began with asking if participants had a hospital visit in the past 12 months and if they responded “No”, the questionnaire would end for them. A “Yes” response entailed them to complete the rest of the third section which gauged information about their previous hospital visit. Within the hospital visit section, the reason for visit (acute condition, chronic condition, emergency reason or a surgery, either emergency or planned), the sector of hospital they visited, perceived certification of physician and type of health-care centre was asked.

The last section consisted of PPE-15 [11], to assess experiences where options of Yes and No were given, and questions were asked regarding tasks completed during the last hospital visit. Each response indicating a negative experience received a score of 1 point, resulting in a cumulative score out of 14.

### Data collection procedure

Prior to the commencement of the main data collection, piloting study was conducted on 40 individuals through face-to- face interviews from the target population in Karachi. This allowed us to assess the feasibility and effectiveness of our data collection tools and questionnaires. Based on the feedback and responses received, necessary adjustments were made to enhance the clarity and simplicity of the questionnaires. Additionally, the Urdu translations were rephrased to make them more colloquial and easily understandable to the participants.

A non-probability convenience sampling technique was followed to recruit and interview participants for our study. The data collection process was conducted in various public places all over Karachi, including malls, parks, hospitals, office buildings, and residential areas. Informed consent was obtained from all participants and the risks and benefits of participation were explained clearly. To ensure the accuracy and consistency of data collection, each data collector underwent comprehensive training before beginning the fieldwork. Data collection was conducted in person, utilizing the Redcap software which allowed online data entry.

Planned Urban Areas: This included planned areas such as parks, supermarkets, and malls [14]. Specifically, for our study, it included locations in East, Central, and South districts of Karachi.

Katchi Abadis: These are unplanned slums often referred to as ‘squatter settlements’, that, over time, grow to form populated settlements [15, 16]. This specifically included underdeveloped areas of Karachi, including Azam Basti and Kharadar.

### Statistical analysis

Categorical variables such as sociodemographic characteristics and previous physician visit information were descriptively represented as frequencies and percentages. The normality of both the expectation and experience scores was assessed using the Shapiro-Wilk test. Continuous variables representing expectation and experience scores were reported using median and interquartile range (IQR).

For regression analysis, both the expectation and experiences scores were converted into binary categorical variables using the median as the cutoff. The expectations outcome was categorized into high expectations and low expectations, with the greater scores being in the high expectation category. While the experience outcome was categorized into negative and positive experiences, with a higher score being in the negative experience category.

The factors associated with high expectations and negative experiences were selected through logistic regression analysis. All covariables with p-values below 0.25 on univariable analysis were included in the subsequent multivariable logistic regression analysis to obtain the adjusted odds ratio (OR). The confidence interval was at 95% and a p-value of < 0.05 was considered statistically significant in all our analyses. Data was analyzed using StataCorp 2017. Stata Statistical Software: Release 15.

### Ethical considerations

The study received approval from Aga Khan University Ethical Review Committee (ERC) under ID#: 2023-8921-25708. Informed consent was obtained from all participants, and their privacy and confidentiality were rigorously protected throughout the research process. Participation in the study was entirely voluntary, and participants were free to withdraw from the study at any time without consequences.

## Results

A total of 424 responses were received through our questionnaire. The median age of respondents was 32 (IQR = 19), ranging from 18 to above 60 years old. Study participant were 60.1% males and 39.9% females. Residents from planned urban areas constituted 73.1% of the overall sample, while 26.9% were from ‘Katchi Abadis’. Most responses (33.5%) were obtained from the Karachi South district. For monthly household income, similar proportions were obtained for all categories with the greatest proportion of respondents earning between 25,000 and 100,000 PKR (38.2%) and the least number of respondents earning greater than or equal to 500,000 PKR (13%). Most of the individuals surveyed had received a bachelor’s degree with 12–14 years of education (31.8%) and only 9.2% of respondents had either no formal education or education till primary school. Employed personnel comprised the greatest proportion of respondents with 63.9% of responses being from them. 113 out of the 424 respondents had a chronic medical condition. Amongst which 10.8% were found to have hypertension and 8.7% had diabetes. These two conditions emerged as the most prevalent chronic diseases in the study sample. All demographic characteristics can be seen in Table 1.

**Table 1:** Sociodemographic characteristics of study participants

	Frequency	%
**Gender**		
Male	255	60.1
Female	169	39.9
**Age**		
18–25	119	28.1
26-40	176	41.5
41-60	114	26.9
More than 60	15	3.5
**Area of Residence**		
Planned Urban Area	310	73.1
Katchi Abadis (slums)	114	26.9
**District**		
Karachi East	138	32.6
Karachi West	34	8.0
Karachi South	142	33.5
Malir	21	5.0
Korangi	9	2.1
Kemari	2	0.5
Karachi Central	78	18.4
**Monthly household income (PKR)**		
≤ 25,000	91	21.5
Between 25,000–100,000	162	38.2
Between 100,000–500,000	116	27.4
≥ 500,000	55	13.0
**Highest level of education**		
< 5 years (Primary school)	39	9.2
5–10 years (Matric/O level)	82	19.3
10–12 years (Intermediate/A level)	61	14.4
12–14 years (Bachelor’s degree)	135	31.8
>14 years (Postgraduate degree)	107	25.2
**Occupational Status**		
Student	61	14.4
Un-Employed (including housewives)	82	19.3
Employed	271	63.9
Retired	10	2.4
**Presence of Chronic Medical Conditions**		
Yes	113	26.6
No	311	73.4

**Fig. 1:**
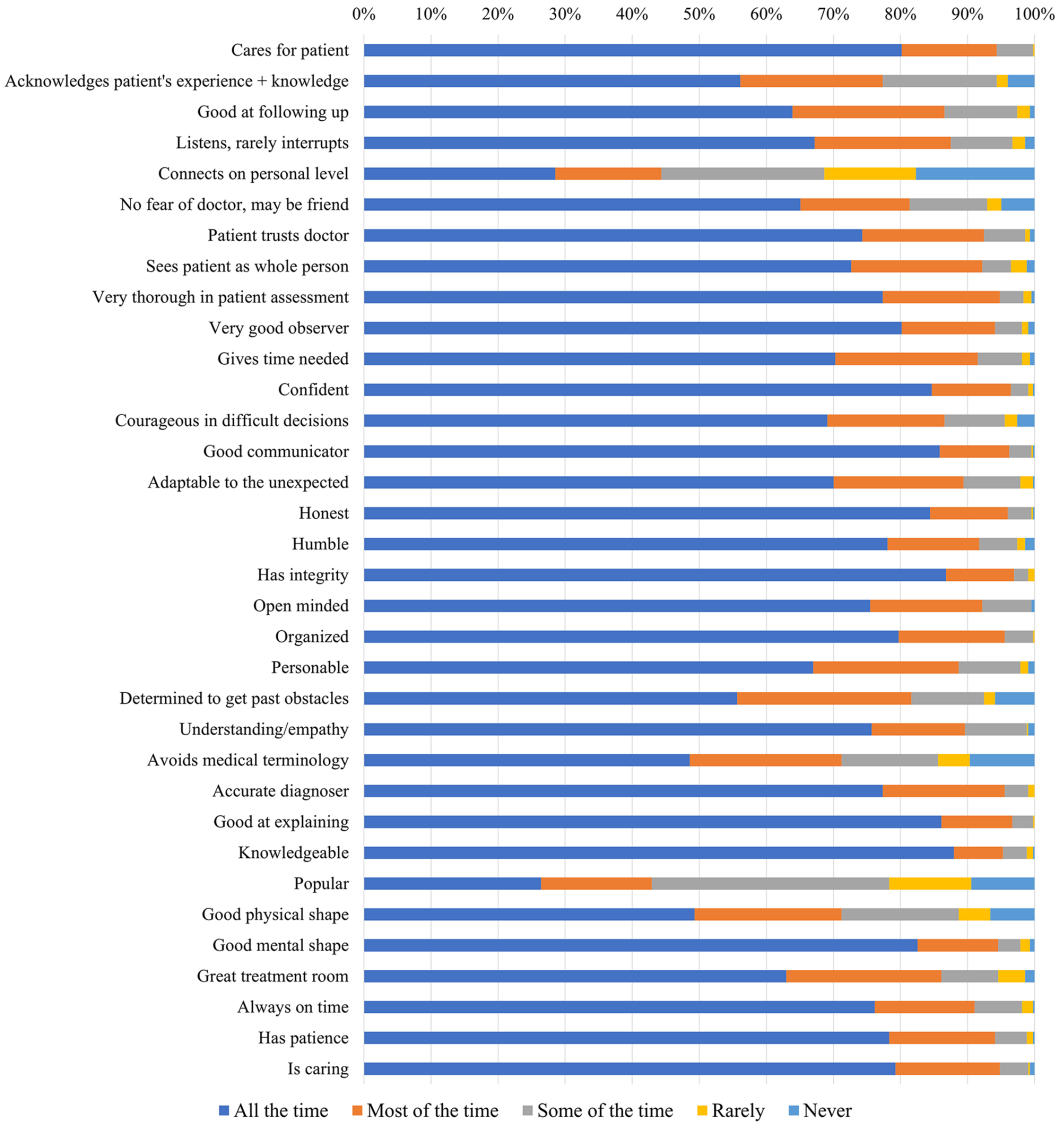
Responses to the modified exceptional good doctor likert scale

The Modified Exceptional Good Doctor Likert Scale was used to assess expectations towards physician responsibilities as seen in Fig. 1. The median score was 30.5 (IQR = 3.3), with a maximum score of 34 and a minimum score of 16. Notable highlights were that a significant majority (80.2%) of respondents valued doctors who demonstrated care for their patients all the time. Additionally, 56.1% emphasized the importance of doctors acknowledging patient experiences and knowledge all the time. The ability to follow up on prior consultations all the time was crucial for 63.9% of participants. Furthermore, 67.2% wanted doctors to not interrupt them during consultations. However, only 28.5% of respondents expected their physician to always establish a personal connection with the patient. Moreover, popularity of the doctor was not valued by most, with the majority (35.3%) of respondents expecting a doctor to be popular only sometimes.

Table 2 provides the results of the logistic regression analysis. In the univariable analysis, several factors were identified as significant predictors of high expectations from physicians. Specifically, being in the age category of adults greater than 60 years of age (OR: 4.65 [1.25–17.3]) as compared to the 18–25 bracket. Residing in squatter settlements (OR: 1.79 [1.15–2.77]) as compared to planned urban settlements. Having a monthly household income either between PKR 100,000 to PKR 500,000 (OR: 0.38 [0.21–0.67]) or greater than PKR 500,000(OR: 0.50 [0.25–0.98]) were also significant as compared to those earning less than PKR 25,000. Lastly, holding a bachelor’s degree (OR: 0.31 [0.14–0.65]) as compared to a primary school education or less was also found to significantly influence expectations. However, upon adjusting for demographic variables (age, area of residence, monthly household income, highest level of education), the results indicated that only age and monthly household income remained significant predictors. Specifically for the age category of adults greater than 60 years (OR: 4.54 [1.18–17.50]) compared to the 18–25 category and the monthly household income between PKR 100,000 to PKR 500,000 (OR: 0.40 [0.20–0.79]) compared to the below PKR 25,000 group, respectively.

**Table 2:** Simple and multivariable logistic regression to assess factors affecting high expectations

Variables	Crude Odds Ratio [95% CI]	Adjusted Odds Ratio [95% CI]
**Gender**		
Male	Reference	–
Female	0.89 [0.60–1.31]	–
**Age**		
18–25	Reference	–
26–40	1.04 [0.65–1.66]	1.03 [0.62–1.72]
18–25 41–60	1.60 [0.95–2.69]	1.66 [0.95–2.91]
More than 60	**4.65 [1.25–17.3]**	**4.54 [1.18–17.50]**
**Area of Residence**		
Planned Urban	Reference	
Squatter Settlement	**1.79 [1.15–2.77]**	1.23 [0.68–2.20]
**Monthly household income (PKR)**		
≤ 25,000	Reference	
Between 25,000–100,000	0.73 [0.43–1.23]	0.81 [0.42–1.43]
Between 100,000–500,000	**0.38 [0.21–0.67]**	**0.40 [0.20–0.79]**
> 500,000	**0.50 [0.25–0.98]**	0.48 [0.22–1.07]
**Highest level of education**		
< 5 years (Primary school)	Reference	
5–10 years (Matric/ O level)	0.52 [0.23–1.15]	0.58 [0.25–1.33]
10–12 years (Intermediate/ A level)	0.56 [0.24–1.31]	0.99 [0.37–2.61]
12–14 years (bachelor’s degree)	**0.31 [0.14–0.65]**	0.53 [0.22–1.28]
>14 years (Postgraduate degree)	0.49 [0.22–1.06]	0.98 [0.38–2.50]
**Occupational Status**		
Student	Reference	–
Un-Employed	1.64 [0.84–3.20]	–
Employed	1.06 [0.61–1.85]	–
Retired	1.10 [0.29–4.20]	–

Significant results are provided in bold

Table 3 shows past hospital visit information. Out of the 424 total respondents, 258 had visited a physician in the past 12 months as a patient. The 258 respondents had visited a physician a median number of 3 times. For 24.4% of the respondents, those visits had been for a chronic condition and amounted to a median visit frequency of two times. 23.3% of previous hospital visits had been for an emergency. The private sector (76%) was most frequently utilized for visiting physicians with primary care centers being the most visited tier visited by 44.6% of the respondents. The vast majority of visits (93%) were conducted with certified physicians.

**Table 3:** Previous physician visit information

Question	Frequency (%)
**Have you visited a physician in the past 12 months as a patient?**	
Yes	258 (60.8)
No	166 (39.2)
**What was the reason for your last visit? (N = 258)**	
Chronic Condition	66 (25.6)
Emergency	55 (21.3)
Acute Condition	133 (51.6)
Surgery	4 (1.6)
**Which sector did your last hospital or clinic visit involve? (N = 258)**	
Public	45 (17.4)
Private	196 (76.0)
Semi-private	17 (6.6)
**Which type of health centre did your last hospital visit involve?**	
Primary	115 (44.6)
Secondary	47 (18.2)
Tertiary	96 (37.2)
**In your last visit, which type of healthcare practitioner do you think you went to? (N = 258)**	
Certified Physician	240 (93.0)
Non-Certified Physician	18(7.0)

**Fig. 2:**
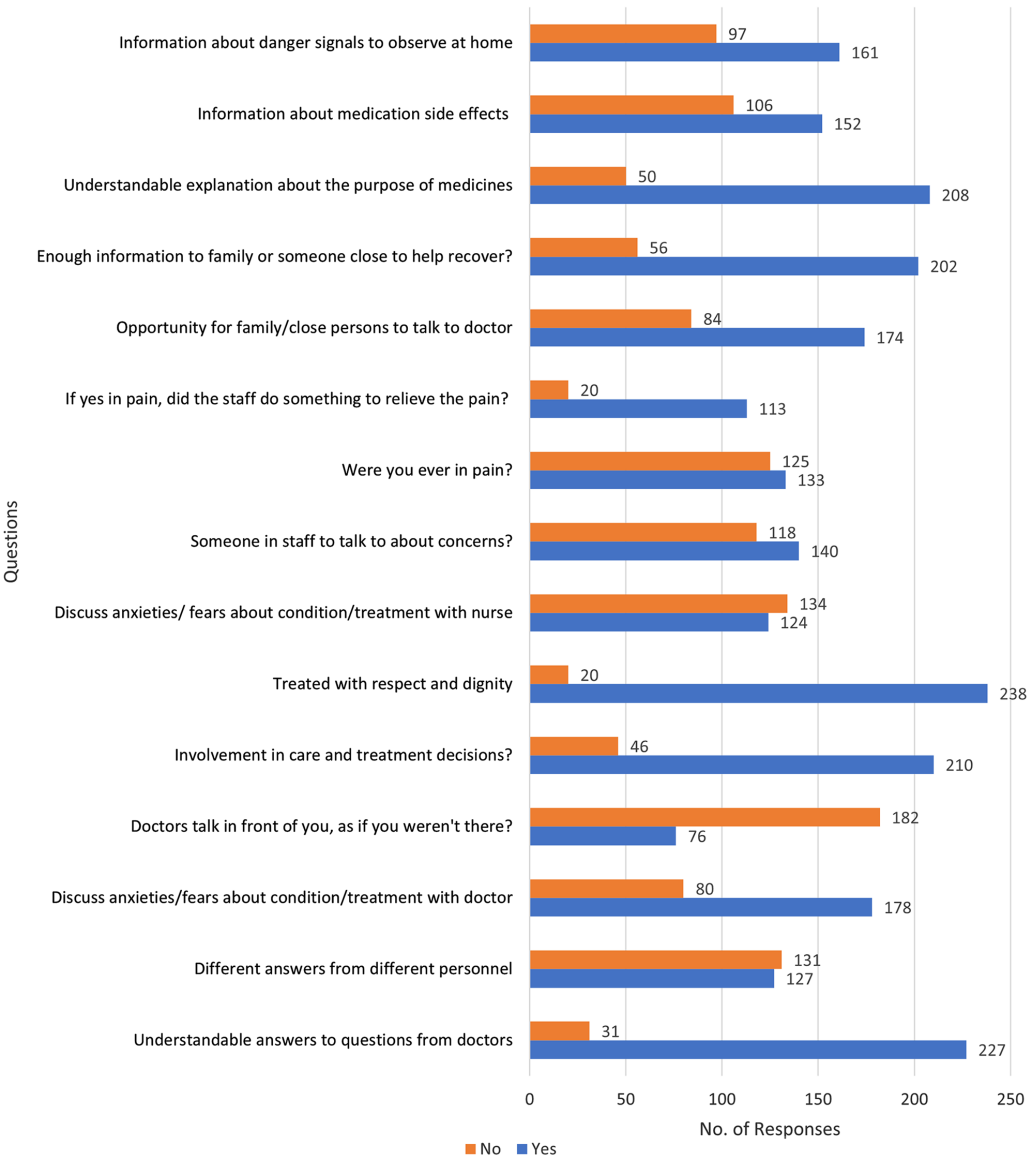
Responses to PPE-15

The 258 individuals who had visited a physician in the past 12 months were subsequently asked about their last physician visit through the Patient Picker Experience Questionnaire, results for which are provided in Fig. 2. The median experience score calculated was 4 (IQR = 4). The maximum score found was a full score of 13 and the minimum score collected was 0. After categorization, a total of 143 negative and 115 positive experiences were found in the study. Notable highlights included that 88% of respondents received understandable answers from doctors for their questions, 69% believed their fears about condition/treatment were discussed and majority felt they were involved in care (81.4%) and were treated with respect and dignity (92.2%). Similar proportions were reported for patients experiencing conflicting answers amongst personnel, 49.2% had experienced it and 50.8% said they had not. For staff-related questions, 54.3% had someone in the staff to talk about their concerns, but 51.9% did not discuss their fears of treatment/condition with a nurse. 51.6% of respondents were in pain during their last visit, out of which 85% experienced relief due to the efforts of the staff.

Table 4 shows simple and multivariable logistic regression with experience as the outcome. Prior to adjustment, greater significant odds of a negative experience were seen with area of residence (OR = 2.40 [1.29–4.43]) and lesser odds with reason for visit and sector visited. Upon adjustment, 69% less likelihood of negative experiences was found with the visit being to a private hospital (OR = 0.31 [0.13–0.70]) compared to a public hospital visit and patients were 62% less likely to report an emergency last visit resulting in negative experience (OR = 0.38 [0.18–0.84]) compared to a chronic condition visit.

**Table 4:** Simple and multivariable logistic regression to assess factors affecting negative experiences

Variables	Crude Odds Ratio [95% CI]	Adjusted Odds Ratio [95% CI]
**Gender**		
Male	Reference	
Female	0.71 [0.43–1.16]	0.93 [0.53–1.61]
**Area of Residence**		
Planned Urban	Reference	
Katchi Abadis (Slums)	**2.40 [1.29–4.43]**	2.31 [0.92–5.76]
**Monthly Household Income (PKR)**		
≤ 25,000	Reference	
Between 25,000–100,000	0.73[0.36–1.51]	1.04 [0.45–2.39]
Between 100,000–500,000	0.61 [0.29–1.29]	1.28 [0.51–3.22]
> 500,000	0.57 [0.24–1.33]	1.12 [0.39–3.22]
**Highest Level of Education**		
< 5 years (Primary school)	Reference	
5–10 years (Matric/ O level)	1 [0.32–3.15]	1.74 [0.51–5.98]
10–12 years (Intermediate/ A level)	0.42 [0.13–1.36]	1.00 [0.25-4.00]
12–14 years (bachelor’s degree)	0.79 [0.27–2.33]	2.49 [0.65–9.61]
>14 years (Postgraduate degree)	0.52 [0.17–1.55]	1.62 [0.41–6.42]
**Last Sector Visited**		
Public	Reference	
Private	**0.34 [0.16–0.70]**	**0.31 [0.13–0.70]**
Semi-private	**0.36 [0.11–0.70]**	0.40 [0.11–1.41]
**Reason for Last Visit**		
Chronic Condition	Reference	
Emergency	**0.41 [0.20–0.85]**	**0.38 [0.18–0.84]**
Acute Condition	0.86 [0.47–1.58]	0.85 [0.45–1.62]
Surgery	0.61 [0.08–4.61]	0.65 [0.08–5.55]
**Presence of Comorbid Condition**		
No	Reference	
Yes	0.84 [0.50–1.42]	

Significant results are provided in bold

## Discussion

Our study marks the first step in generating Pakistani-context specific recommendations to tailor healthcare towards patient expectations by assessing public expectations from physicians and evaluating experiences to determine where the gaps in practice exist. We found the public to have generally high expectations from physicians but mixed experiences, with negative experiences outnumbering positive ones. Factors associated with high expectations included older age group and lower monthly income while factors associated with negative experiences included sector visited and reason for visit.

These results corroborate those of the previously conducted “Good Doctor Survey”, suggesting that the qualities desired in an exceptional doctor match with the Pakistani publics expectations [12]. A median score of 30.5 out of 34 was observed for expectations from physician responsibilities pointing towards high expectations amongst the public towards physicians. This can be attributed to the elevated status the public assigns to a physician in Pakistan, as even national studies have proclaimed that the majority of the Pakistani population trust healthcare professionals for advice relating to their health [17].

In our study, age was a factor affecting expectations, with adults above 60 years of age having greater odds of higher expectations than younger adults age groups. This difference could be attributed to the relatively high prevalence of ailments requiring routine follow-ups, such as chronic conditions, which make a close patient-physician relationship vital due to the increased frequency of visits [18, 19]. Due to multiple comorbidity and frailty, each clinical insult often adds to the risk of adverse outcomes in the geriatric population, emphasizing the need for specialized care [20]. The most optimal first step to specialized care can be to consider matching patient expectations. Another study from Karachi in 2009 found an inverse relation between patient satisfaction and frequency of health complaints, thereby indicating a greater need to meet expectations for older age groups [19].

Furthermore, our study found that lower-income households had greater odds of higher expectations, especially with 41.2% of the population in the PKR 25,000-100,000-income bracket reporting high expectations. Although further studies need to be conducted to shed more light on this association, it is plausible that the country’s healthcare financing system plays a role in this finding. As our study found, about 52.4% of the participants were not receiving any form on healthcare insurance. This coupled with the extremely high prevalence of out-of-pocket expenditure even at public healthcare level, disproportionately affects the low-income groups [21, 22]. Hence, high expectations from the healthcare sector might be a consequence of the massive impact of healthcare costs on their income percentage and with the public wanting their healthcare experience to reflect the price paid for it.

Previous research has highlighted the need for more studies diving into the insights derived from patient-reported experience measures [23]. Therefore, we used the Patient Picker Experience questionnaire to assess physicians’ performance from the perspective of those receiving care and obtained a median score of 4 out of the total 14. With lower scores signifying positive experiences, overall, many individuals reported a positive experience with the healthcare system, but some unique trends were also identified. It was praiseworthy to find many physicians providing concise explanations to patients regarding the purpose and potential side effects of prescribed medications. This practice can be attributed to efforts by physicians to address the prevalent issue of medical mistrust within the Pakistani population, as exemplified by the widespread skepticism towards vaccines observed across the country [24]. Therefore, building interpersonal trust between physicians and patients could be one of the many steps forward eradicating medical mistrust within Pakistan.

Greater odds of negative experience were only found with residents of Katchi Abadis, consistent with the evidenced disparities within slum areas [25]. Our study found most participants to experience respect and dignity in their consultation. This positive finding represents a match in expectations and experiences, as a 2005 study also found autonomy and respect to be a common expectations amongst Pakistani patients [26] This serves to show that the paternalistic medical practice which has existed in Pakistan for a long time may finally be headed in the direction of patient-centeredness [27].

Respondents with their last medical visit being for an emergency had lesser odds of having a negative experience as compared to those visiting for chronic conditions [28]. Although older literature based on patient perspectives has pointed out long waiting times, inadequate privacy and lack of information have often been reasons for dissatisfaction in Pakistani hospitals [29]. This could likely be due to the recent interventions to improve emergency care in Pakistan [28, 30]. Nonetheless, the increased chances of a chronic visit being a negative experience could be due to the widespread burden of non-communicable diseases in Pakistan leading to more frequent visits, and thereby exhausting the patient or due to the lackluster quality of care provided for such ailments [31, 32]. Regardless, more studies need to be conducted to evaluate reasons for negative experiences amongst individuals visiting for chronic care.

In our study, there were greater odds of a public healthcare facility visit resulting in a negative experience compared to private and semiprivate settings. This could also be attributable to the fact that the volume of patients in the private sector is less, thus allowing doctors spend more time with their patients leading to a better patient experience [33]. A study carried out in a private sector in India revealed that patients were satisfied with the adequate time provided for consultation by the doctor and explanation of medical concepts provided by the physician in an understandable manner [34]. Therefore, policies that address public sector disparities are needed especially with equity being at the forefront of improving health care delivery [35].

Our study was only conducted in Karachi, therefore limiting generalizability of the results for national and regional level interventions, thus nationwide studies need to be conducted for policy change at the national level to implement patient-centered care. Furthermore, patient expectations are a highly subjective matter with a lot of nuances involved which a quantitative study design cannot accommodate. Additionally, the PPE-15 tool falls short in exploring several crucial aspects of patient expectations, such as waiting time, consultation fees, and satisfaction with prescribed drugs and tests. These unexplored factors have been demonstrated to hold significant importance in a patient’s physician encounter, particularly from the perspective of a resident of Katchi Abadis, as evidenced by previous literature [36]. Regardless, our study is the sole source of expectations and experiences assessed in tandem and may be the only study targeting the public from Pakistan.

Patient-centered care is crucial for achieving optimal outcomes but remains a novel concept in developing countries like Pakistan [29, 37]. As part of their professional responsibility, clinicians need to be mindful of these patient expectations and should adapt their practice to fulfill them [38]. Unfortunately, despite training that recommends otherwise, physicians sometimes tend to overlook their patients’ expectations and concerns [39]. Therefore, in view of our findings, we recommend that doctors actively implement strategies to facilitate open dialogue with their patients [40]. On the other hand, instead of insisting that physicians accomplish more during the short time frame they have with the patient, the public should consider more realistic expectations such as better management of resources within their reach [41].

## Conclusions

Bridging the gap between expectations and experiences of the public is vital for patient-centered healthcare. High expectations exist amongst the public regarding physician role and responsibilities with age and household income being significant factors affecting expectations. At the same time, the public, reporting a mixture of experiences, the type of sector visited, and the reason for visit are significant determinants of their experience. Our study therefore provides recommendations applicable to all LMICs which face similar health disparities and therefore recommends the public to view physicians as humans who are not perfect beings and for physicians to acknowledge their responsibilities to cater patient expectations and provide excellent experiences.

### Electronic supplementary material

Below is the link to the electronic supplementary material.

**Supplementary Material 1:** Study Questionnaire

## Data Availability

The dataset and its associated materials are available from the corresponding author upon reasonable request.

## Abbreviations

ERC: Ethical review committee
OR: Odds ratio
IQR: Interquartile range
WHO: World health organization
LMICs: Low- and middle-income countries
PPE-15: Patient picker experience-15
