# Supplementary material for: An assessment of public experiences and expectations with physicians: a cross sectional study from Karachi, Pakistan
Source: BMC Health Serv Res. 2024 Jan 18;24:108. doi: 10.1186/s12913-023-10519-2 (PMC10797951; doi:10.1186/s12913-023-10519-2)
Supplement: Supplementary file 1 — Supplementary Material 1: Study Questionnaire [file 12913_2023_10519_MOESM1_ESM.pdf]

An Assessment of Public Experiences and Perceptions towards Physician Responsibilities: A Cross Sectional Study from Karachi, Pakistan

Supplementary File 1: Questionnaire

Section 1: Sociodemographic

1. What is your age in years?
2. What is your gender?
    a. Male
    b. Female
    c. Other
3. What is your area of residence?
    a. Urban
    b. Slums/Katchi Abadi
4. What district is your residence located in?
    a. Karachi East
    b. Karachi West
    c. Karachi South
    d. Malir
    e. Korangi
    f. Kemari
    g. Karachi Central
5. What is your monthly household income (PKR)
    a. ≤ 25,000
    b. Between 25,000 – 100,000
    c. Between 100,000 – 500,000
    d. ≥ 500,000
6. What is your highest level of education?
    a. <5 years (Primary school)
    b. 5-10 years (Matric/ O level)
    c. 10-12 years (Intermediate/ A level)
    d. 12-14 years (bachelor’s degree)
    e. >14 years (Postgraduate degree)
    f. No formal education
7. What is your current occupation?
    a. Student
    b. Un-Employed
    c. Employed
    d. Retired
8. What, if any, of the chronic medical conditions do you currently have
    a. Diabetes

b. Hypertension
c. Asthma
d. Tuberculosis
e. Chronic obstructive pulmonary disease (COPD)
f. Cardiovascular diseases (Coronary artery disease, Heart failure)
g. Chronic kidney disease
h. Depression
i. Autoimmune diseases
j. Cancer
k. Other

9. If selected ‘Other’, please specify.

Section 2: Modified Exceptional Good Doctor Likert Scale

1. Do you think the doctor should care for the patient?
   a. All the time
   b. Most of the time
   c. Sometimes
   d. Rarely
   e. Never
2. Do you think the doctor should acknowledge patient’s experience and knowledge?
   a. All the time
   b. Most of the time
   c. Sometimes
   d. Rarely
   e. Never
3. Do you think the doctor should be good at following things up or addressing items from prior consultation?
   a. All the time
   b. Most of the time
   c. Sometimes
   d. Rarely
   e. Never
4. Do you think the doctor should listen well?
   a. All the time
   b. Most of the time
   c. Sometimes
   d. Rarely
   e. Never
5. Do you think the doctor should connect with the patient on a personal level?
   a. All the time
   b. Most of the time
   c. Sometimes
   d. Rarely
   e. Never

6. Do you think the patient should not have any fear of the doctor and may see him as a friend?
   a. All the time
   b. Most of the time
   c. Sometimes
   d. Rarely
   e. Never
7. Do you think the patient should trust the doctor?
   a. All the time
   b. Most of the time
   c. Sometimes
   d. Rarely
   e. Never
8. Do you think the doctor should see the patient as a whole person not just a collection of symptoms?
   a. All the time
   b. Most of the time
   c. Sometimes
   d. Rarely
   e. Never
9. Do you think the doctor should be very thorough in the patient's assessment?
   a. All the time
   b. Most of the time
   c. Sometimes
   d. Rarely
   e. Never
10. Do you think the doctor should be a good observer?
    a. All the time
    b. Most of the time
    c. Sometimes
    d. Rarely
    e. Never
11. Do you think the doctor should give the patient the time needed?
    a. All the time
    b. Most of the time
    c. Sometimes
    d. Rarely
    e. Never
12. Do you think the doctor should be confident?
    a. All the time
    b. Most of the time
    c. Sometimes
    d. Rarely
    e. Never
13. Do you think the doctor should be courageous when making difficult decisions?
    a. All the time

b. Most of the time
c. Sometimes
d. Rarely
e. Never

14. Do you think the doctor should be good at communicating?
    a. All the time
    b. Most of the time
    c. Sometimes
    d. Rarely
    e. Never
15. Do you think the doctor should be adaptable i.e. respond to the unexpected?
    a. All the time
    b. Most of the time
    c. Sometimes
    d. Rarely
    e. Never
16. Do you think the doctor should be honest?
    a. All the time
    b. Most of the time
    c. Sometimes
    d. Rarely
    e. Never
17. Do you think the doctor should be humble?
    a. All the time
    b. Most of the time
    c. Sometimes
    d. Rarely
    e. Never
18. Do you think doctors should have integrity?
    a. All the time
    b. Most of the time
    c. Sometimes
    d. Rarely
    e. Never
19. Do you think doctors should be open minded?
    a. Completely disagree
    b. Disagree
    c. Neutral
    d. Agree
    e. Completely agree
20. Do you think doctors should be organized?
    a. All the time
    b. Most of the time
    c. Sometimes
    d. Rarely
    e. Never

21. Do you think the doctor should be personable?
    a. All the time
    b. Most of the time
    c. Sometimes
    d. Rarely
    e. Never
22. Do you think the doctor should be determined to get past bureaucratic obstacles that affect treatment?
    a. All the time
    b. Most of the time
    c. Sometimes
    d. Rarely
    e. Never
23. Do you think the doctor should be understanding and/or shows empathy?
    a. All the time
    b. Most of the time
    c. Sometimes
    d. Rarely
    e. Never
24. Do you think the doctor should avoid using medical terminology the patient doesn't understand?
    a. All the time
    b. Most of the time
    c. Sometimes
    d. Rarely
    e. Never
25. Do you think the doctor should be accurate in diagnosing the issue/problem?
    a. All the time
    b. Most of the time
    c. Sometimes
    d. Rarely
    e. Never
26. Do you think the doctor should be good at explaining things?
    a. All the time
    b. Most of the time
    c. Sometimes
    d. Rarely
    e. Never
27. Do you think the doctor should be knowledgeable?
    a. All the time
    b. Most of the time
    c. Sometimes
    d. Rarely
    e. Never
28. Do you think doctors should be popular?
    a. All the time

b. Most of the time
c. Sometimes
d. Rarely
e. Never

29. Do you think the doctor should be in good physical shape?
    a. All the time
    b. Most of the time
    c. Sometimes
    d. Rarely
    e. Never
30. Do you think the doctor should be in good mental shape?
    a. All the time
    b. Most of the time
    c. Sometimes
    d. Rarely
    e. Never
31. Do you think the doctor should be in an especially harmonious or care for treatment room?
    a. All the time
    b. Most of the time
    c. Sometimes
    d. Rarely
    e. Never
32. Do you think the doctor should be on time?
    a. All the time
    b. Most of the time
    c. Sometimes
    d. Rarely
    e. Never
33. Do you think the doctor should have patience?
    a. All the time
    b. Most of the time
    c. Sometimes
    d. Rarely
    e. Never
34. Do you think the doctor should be caring?
    a. All the time
    b. Most of the time
    c. Sometimes
    d. Rarely
    e. Never

Section 3: Hospital Visit Information

1. Have you visited a physician in the past 12 months as a patient?
   a. Yes
   b. No

If the answer to Q1 was No, the survey ends here for this participant.

2. How many times have you visited the physician in the past 12 months as a patient?_______
3. Have your physician visits been for any chronic condition?
   a. Yes
   b. No
4. If yes, how many? ______
5. Have any of your visits been for an emergency situation?
   a. Yes
   b. No
6. If yes how many? ______
7. What was the reason for your last visit?
   a. Chronic
   b. Emergency
   c. Acute
   d. Surgery
8. Which sector did your last hospital or clinic visit involve?
   a. Public
   b. Private
   c. Semi-private
9. Which type of health center did your last hospital visit involve?
   a. Primary
   b. Secondary
   c. Tertiary
10. In your last visit, which type of healthcare practitioner did you go to?
    a. Certified physician
    b. Compounder/ Dispenser
    c. Hakeem
    d. Faith healer
    e. Homeopathic doctor
    f. Any other traditional healer (mention_____)

Section 4: Patient Picker Experience-15

Please answer the following questions with your experience as a patient on your most recent visit.

1. Understandable answers to questions from doctors
    a. Yes
    b. No
2. Different answers from different personnel
    a. Yes
    b. No
3. Discuss anxieties/fears about condition/treatment with doctor.
    a. Yes
    b. No
4. Doctors talk in front of you, as if you weren't there?
    a. Yes
    b. No
5. Involvement in care and treatment decisions?
    a. Yes
    b. No
6. Treated with respect and dignity.
    a. Yes
    b. No
7. Discuss anxieties/ fears about condition/treatment with nurse.
    a. Yes
    b. No
8. Someone in staff to talk to about concerns?
    a. Yes
    b. No
9. Were you ever in pain?
    a. Yes
    b. No
10. If yes, did the staff take action to relieve the pain.
    a. Yes
    b. No
11. Opportunity for family/close persons to talk to doctor.
    a. Yes
    b. No
12. Enough information to family or someone close to help recover.
    a. Yes
    b. No

13. Understandable explanation about the purpose of medicines
    a. Yes
    b. No
14. Information about medication side effects
    a. Yes
    b. No
15. Information about danger signals to observe at home.
    a. Yes
    b. No

______END OF QUESTIONNAIRE___
